# Discrete Element Study on Bending Resistance of Geogrid Reinforced Cement-Treated Sand

**DOI:** 10.3390/ma16072636

**Published:** 2023-03-26

**Authors:** Hao Luo, Xuan Wang, Yu Zhang, Jiasheng Zhang

**Affiliations:** Department of Civil Engineering, Central South University, 68 Shaoshan South Road, Changsha 410075, China

**Keywords:** cement-treated sand, geogrid reinforcement, three-point bending tests, meso-mechanism

## Abstract

Cement-treated sand reinforced with geogrids (CTSGs) has higher bending resistance and toughness than cement-treated sands (CTSs). To explore the reinforcement mechanism of geogrids with different stiffness and layers on CTSGs, three-point bending tests and numerical tests based on DEM are carried out on CTS specimens and CTSG specimens considering different reinforcement conditions. The results show that the geogrids and cement-treated sands have good cooperative working performance. Compared with CTSs, CTSG specimens show better ductility, flexural strength and toughness. The increase in geogrid stiffness and geogrid layers promote the reinforcement effect. On the meso-level, different geogrid stiffness and layers affect the crack propagation speed and distributions of cracks due to the anchorage action of geogrids, resulting in different reinforcement effects. In addition, the layers and stiffness of geogrids affect the evolution of the internal force chains of CTSG specimens. Both the increase in geogrid layers and decrease in geogrid stiffness reduce the average internal force of geogrids and weaken the anisotropy of the normal contact force of the specimens. The simulation results interpret the reinforcement mechanism of a CTSG specimen from crack development and internal force evolution, which can support a mesoscopic supplement to laboratory tests.

## 1. Introduction

Classic engineering materials such as cement-treated sand and concrete have high compressive strength but poor flexural and bending resistance, often requiring reinforcing materials with good tensile properties. On the other hand, with the increasing awareness of environmental protection and advances in production and construction technology, geogrids have been widely used in civil engineering projects [1]. Compared to other reinforcement materials, geogrids not only possess a higher specific strength (the ratio between strength of the material and its apparent density), but also have advantages such as green economy and corrosion resistance, which allow them to be used in thin cross-sectional layers such as those in highway pavements or in corrosive environments with high ion concentrations [2]. In recent years, environmental protection requirements for infrastructure construction have been increasing, and the application of geogrids in constructions meet the requirements of the green economy, making them suitable for existing infrastructure maintenance, ecological restoration and other fields [2,3]. Therefore, studying the flexural performance of geogrid-reinforced cement mortar materials has practical significance.

Currently, laboratory testing is the main method for studying the mechanical properties of geogrid-reinforced cement mortar materials. Based on a series of laboratory tests, researchers have found that geogrid reinforcement can effectively delay the propagation and development of micro-cracks [2,4], significantly improving the flexural capacity of the components. The position and type of external loads applied, as well as the location and number of layers determined by the geogrid reinforcement design [4,5,6,7,8,9], will significantly impact the overall performance of cement-treated sand reinforced with geogrids (CTSGs). Some researchers have pointed out that matrix and reinforcement properties (including cement type, sand and gravel particle size distribution, geogrid type, etc.) are also important reference factors for engineering application design [6,10]. Among them, stiffness, as an important physical parameter of materials, can significantly affect the overall properties of composite materials. However, in geogrid-reinforced cement mortar research, due to the difficulty in controlling stiffness variables in experiments, there are fewer quantitative analyses of geogrid stiffness factors.

Numerous laboratory tests have demonstrated the feasibility and advantages of geogrid-reinforced cement mortar [4,5,6,7,8,9], but laboratory tests still have their limitations. With the development of discrete element numerical simulations, researchers have used numerical experiments to compensate for the shortcomings of laboratory tests, such as the difficulty in controlling variables and inability to conduct microscopic studies. The Discrete Element Method (DEM) was first proposed by Cundall and Strack in 1979 and has since been applied by many to study rock mechanics [11]. The results show that the DEM can effectively simulate the crack generation and evolution process in the interlocking particles of rock material [12]. Similarly, concrete can also be considered a bonded particle material and can be studied through DEM simulations. Lee et al. used the DEM model to simulate rock failure behavior [13], while Xin Tan et al. simulated the failure process of recycled aggregate concrete and natural aggregate concrete using the DEM, and identified the weak parts of the concrete specimens through the evolution of micro-cracks [14]. P. Wang et al. calibrated the DEM model parameters using uniaxial compression and Brazilian splitting tests and studied the crack propagation mechanism of concrete specimens [15]. It is evident that researchers are able to study the internal mechanisms that are difficult to observe in laboratory experiments by using numerical techniques to conduct research from the macro to micro level. Through DEM simulations of reinforced cement mortar with geogrids, the reinforcing mechanism of geogrids in cement mortar can be systematically analyzed from a microscopic perspective. However, there is currently limited research on DEM simulations of geogrid-reinforced cement mortar.

In summary, to enhancing the understanding of the CTSG structure from the macro and micro perspectives, this paper establishes a discrete element numerical model of CTSG specimens based on data obtained from laboratory three-point bending tests and analyzes the effects of geogrid stiffness and reinforcement layer number on the load deflection relationship, flexural strength and toughness of CTSGs. The microscopic analysis of different geogrid stiffness factors quantitatively is presented.

## 2. Laboratory Test

### 2.1. Material and Methods

**Figure 1:**
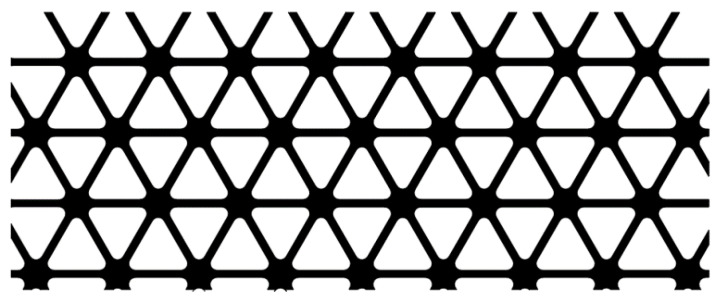
Triaxial geogrid in laboratory test.

**Figure 2:**
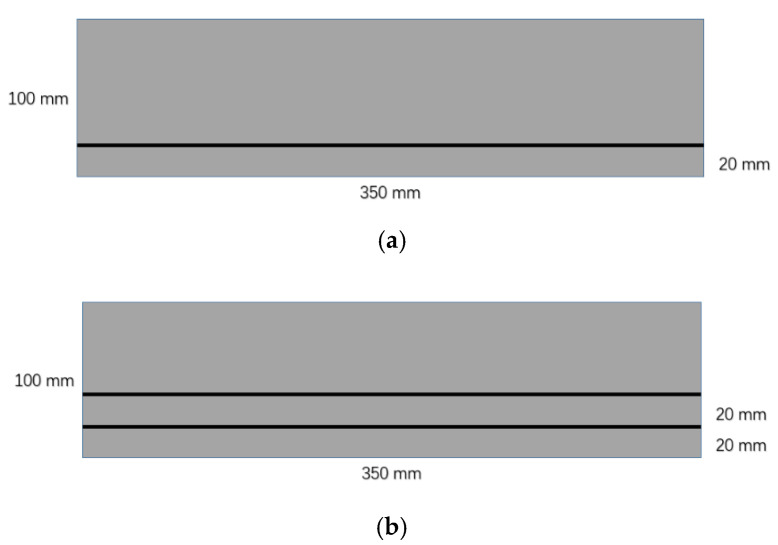
Schematic diagram of cement-treated sand reinforced with geogrids in laboratory test. (**a**) Single-layer geogrid CTSG specimen. (**b**) Double-layer geogrid CTSG specimen.

The materials selected in the test mainly include sand (standard construction sand), cement (P.O cement) and triaxial geogrid (as shown in Figure 1). The density of the geogrid used is 1350 kg/m^3^ and the thickness is 3 mm. The schematic diagram of the working condition with different geogrid layers is shown in Figure 2.

The laboratory bending test of CTSG specimens follows ASTM C1609/C1609M [16]. In order to ensure the correctness of the test results, each group of tests was repeated twice.

### 2.2. Results and Discussion

In order to evaluate the mechanical properties of CTSG specimens, the flexural strength index f and toughness index are introduced according to ASTM C1609/C1609M-10 [16]. The calculation formula of bending strength *f* is as follows:

(1) $$f=\frac{PL}{bd^{2}}$$

where f is bending strength, kPa; *P* is the corresponding required load value, kN; *L* is the span length, m; *b* is the width of the specimen, m and *d* is the thickness of the specimen, m.

Toughness refers to the area enclosed by the deflection–load curve of the material, which can effectively reflect the energy absorption capacity and dynamic load resistance of the material. The calculation formula is as follows:

(2) $$T_{x_{1}}^{D}=\int_{0}^{x_{1}}F\left(x\right)dx$$

In the formula, *F*(x) is load–deflection curve.

According to ASTM C1609/C1609M-10 [16], the first peak strengths *P*_1_ of the key node are *P*_1_ (the first obvious nonlinear point of the load–deflection curve, the corresponding load value is called *P*_1_, and the corresponding deflection value is called *δ*_1_, corresponding to the bending strength *f*_1_) and deflection *L*/150 (corresponding to the residual strength *f*_2_).

**Figure 3:**
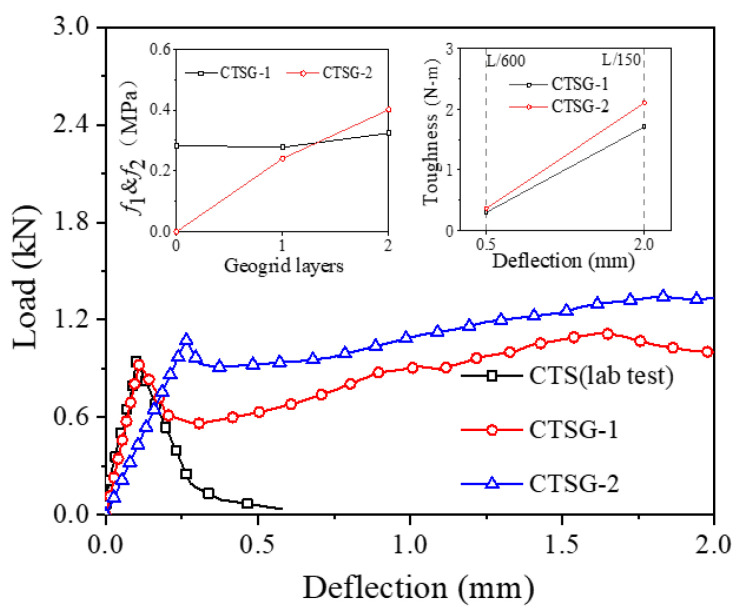
The evolution of deflection with load and the variation of the bending strength and toughness in three-point bending tests.

Figure 3 shows the load–deflection curves of specimens with different reinforcement layers and summarizes the toughness and flexural strength under different working conditions. As can be seen from the figure, during the initial stage of loading (before the deflection reaches the first peak point *P*_1_), the mechanical responses of the specimens under different working conditions are similar. The loads both experience linear increases until the deflection increase to *P*_1_, then the load drops rapidly from point P1 onwards. However, the load of the CTS specimen decreases sharply to 0 after *P*_1_, while the loads of the CTSG specimens increase after decreasing, which illustrates that the reinforced specimens exhibit better bearing capacity. In addition, the bearing capacity of the double-layer reinforced condition is better than that of the single-layer reinforced condition. Comparing the bending strength index *f* under different geogrid layers, it can be seen that the bending strength f1 changes slightly with the increase in the number of layers, while *f*_2_ increases significantly. The CTS specimen, due to its quick fracturing, fails to demonstrate any toughness. For the geogrid-reinforced specimens, the rate of toughness increase (slope of the toughness–deflection curve) for the double-layer reinforced specimens is faster than that of the single-layer reinforced specimens.

## 3. Simulated Test

In order to investigate the influence of geogrid stiffness and layer number on the mechanical response of CTSGs, this investigation uses discrete element software PFC^2D^ to simulate the three-point bending test of the CTS and CTSG specimens with different geogrid stiffness and layer number, and analyzes the reinforcement mechanism and deformation failure mechanism of geogrid on the bending resistance of the CTSGs.

### Numerical Modeling

In order to ensure that the numerical test is consistent with the laboratory test, the numerical test needs to consider the cohesion of cement mortar, the tensile effect of the grid and the interaction between the mortar and the grid. There are two main modeling methods for discrete element simulation of bonding materials such as cement. One is to simplify the cement mortar into an aggregate of spherical particles and consider its bonding through a parallel bonding contact model [17,18]. The second is to simulate cohesion by generating cement particle bonding with sand particles [19].Considering that the size of sand used in the test is small, the simulation efficiency using the second method is extremely low; thus, the first method is used in this simulation. In order to make the numerical model generate enough contact to ensure particle bonding, the matrix porosity is 4 %. In order to prevent soil particles from passing through the gaps in the geogrid after it has been stretched, continuous bonded balls are adopted to simulate the geogrid with a certain amount of overlap between the bonded particles (the ratio of the spacing of center of adjacent balls to the ball diameter is 0.72).

**Figure 4:**
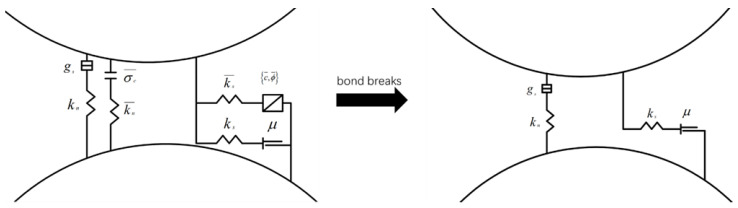
Parallel bond model.

The parallel bond model used in the simulation can transfer bending moment and resist rotation, as shown in Figure 4 When the parallel bond between particles is broken, the model degrades into a linear contact model, as shown in Figure 4, allowing particles to rotate and slide relative to each other. Previous investigations [17,18] have shown that the particle discrete element model based on the parallel bond model can reproduce various mechanical properties of concrete materials.

The bond stress in the parallel bond model is determined by the correlation between force and displacement. This correlation is established by considering various parameters such as normal and tangential stiffness, tensile and shear strength and bond radius factor, etc. The force and force moment acting on the parallel bond can be denoted as $F_{i}$ and $M_{i}$, respectively. These forces are made up of components that act in both normal and tangential directions, and can be represented as:

$$\bar{F_{i}}=\bar{F_{i}^{n}}+\bar{F_{i}^{s}}$$

$$\bar{M_{i}}=\bar{M_{i}^{n}}+\bar{M_{i}^{s}}$$

The vectors $\bar{F_{i}^{n}}$ and $\bar{M_{i}^{n}}$ represent normal force and moment, while $\bar{F_{i}^{s}}$ and $\bar{M_{i}^{s}}$ represent tangential force and moment, respectively. When the parallel bond is established, both $F_{i}\;$ and $\;M_{i}$ will be set to zero. The forces and moments caused by further relative displacement and rotation will be added to the current values. These forces and moments resulting from relative displacement and rotation can be expressed as.

$$\{\begin{array}{c} \Delta \bar{F_{i}^{n}}=-k^{n}A\Delta u_{i}^{n}\\ \Delta \bar{F_{i}^{s}}=-k^{s}A\Delta u_{i}^{s}\\ \Delta \bar{M_{i}}=-k^{n}I\Delta \theta_{i} \end{array}$$

The variables used in the equation include $\Delta u_{i}=v_{i}\Delta t$, $\Delta \theta_{i}=\omega_{i}\Delta t$, $A=2\bar{R}$, and $I=\left(2/3\right){\bar{R}}^{3}$. $\Delta \bar{F_{i}^{n}}$ and $\Delta \bar{F_{i}^{s}}$ represent the increment in the normal and tangential bonding force, respectively, while $\Delta \bar{M_{i}}$ represents the force moment increment. $k^{n}$ and $k^{s}$ are the normal and tangential stiffness, respectively, and A is the bonding area. $\Delta u_{i}^{n}$ and $\Delta u_{i}^{s}$ are the displacement increments in the normal and tangential directions, respectively, and $\Delta \theta_{i}$ represents the relative rotation angle of contact particles. I represents the inertia moment of the bonding surface on the neutral axis,$\;v_{i}$ is the relative velocity of contact particles, $\bar{R}$ is the contact bond radius and Δ*t* represents time steps. The parallel bond experiences the maximum tensile stress and shear stress.

$${\bar{\sigma }}_{i_{max}}=\frac{{\bar{F}}_{i}^{n}}{A}+\frac{\left|{\bar{M}}_{i}^{n}\right|\bar{R}}{I}$$

$${\bar{\tau }}_{i_{max}}=\frac{\left|{\bar{F}}_{i}^{n}\right|}{A}$$

If the maximum tensile stress acting on the bond is greater than the ultimate tensile strength of the bond itself, the bond will break and produce tensile cracks. Similarly, if the maximum shear stress acting on the bond exceeds the ultimate shear strength of the bond itself, the bond will break and produce shear cracks.

**Figure 5:**
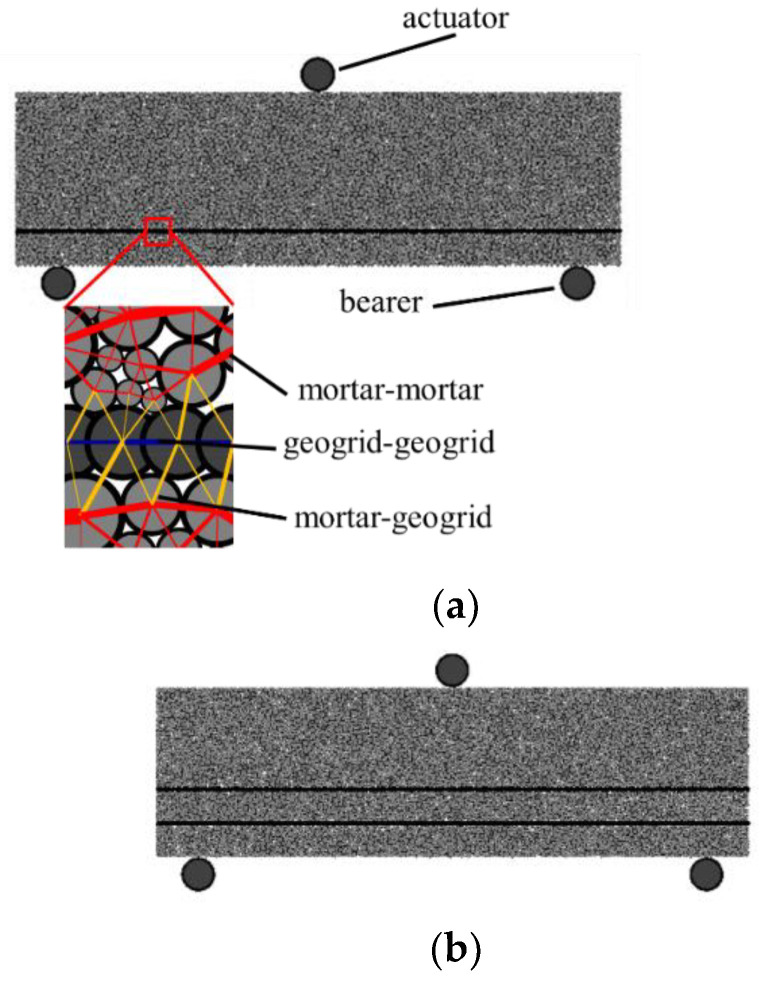
Model of three-point bending test in DEM simulation. (**a**) Single-layer geogrid CTSG specimen. (**b**) Double-layer geogrid CTSG specimen.

The numerical test process is as follows: firstly, a rectangular closed area is generated according to the actual size of 350 mm × 100 mm, and the particles are randomly placed according to the target porosity and particle size range (2–3 mm uniform gradation) of the specimen. In the process of specimen generation, the contact model between the particles and wall is set as the linear elastic model, and the friction coefficient between particles is set as 0, so as to ensure that the particles can be evenly and densely accumulated. The damping coefficient is 0.7, and then the internal force balance is calculated. Based on the position of the geogrid during the test, single-layer and double-layer geogrids were generated by clumps. After calculating the balance, the geogrid was replaced by clusters, and the solving operation was completed until the unbalanced force ratio was less than 1 × 10^−5^. According to the actual loading situation of the three-point loading test, the clump simulation actuator and bearers are created according to the response size and spacing, as shown in Figure 5 The single-layer reinforced CTSG and particle contact relationship are shown in Figure 5a and the double-layer reinforced CTSG is shown in Figure 5b.

In order to reduce the influence of inertia force in the process of numerical simulation, the method of slowly increasing the loading speed is adopted when the simulated sample is loaded: after the loading rod contacts the specimen, it is slowly accelerated from 0 mm/min to a predetermined loading speed of 0.05 mm/min through 50,000 cycles. When the loading rod displacement is 2.1 mm (>*L*/150 mm), the loading ends.

In order to ensure the reliability of the numerical test results, the mesoscopic parameters of the discrete element model are calibrated based on the laboratory test results. According to the selection of the contact model above, firstly, the stiffness and parallel bond stiffness of the cement mortar are calibrated according to the laboratory bending test results of the CTS specimens, and then the stiffness and parallel bond stiffness of the geogrid are calibrated based on this. The calibrated parameters of the cement mortar and geogrid are applied to the bending simulation test of double-layer CTSG specimens. When the double-layer CTSG specimens can still greatly reflect the mechanical response in the laboratory test, the parameters are considered reasonable.

**Figure 6:**
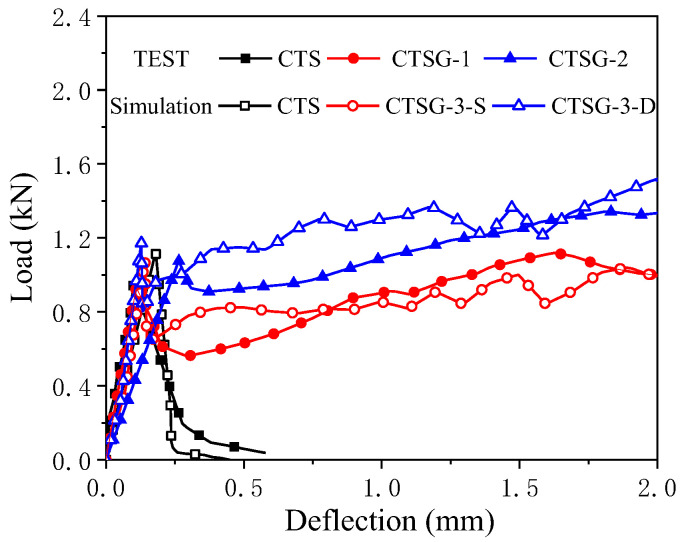
Comparison of simulation and test results.

Figure 6 is the comparison of numerical test and laboratory test results. It can be seen from the figure that for CTS specimens and single-layer CTSG specimens, the simulation results can well match the load-deflection response of the test. For the double-layer CTSG specimen, the initial stiffness of the simulation results is greater than the test results (affected by the sample preparation deviation caused by multi-layer reinforcement and the simplified simulation), while the peak strength and the mechanical properties after the peak are basically the same. It can be considered that the results of the numerical simulation are similar to those of the laboratory test, and the selected model parameters and simulation results are reliable.

Three different geogrids stiffnesses, in which the values are normal [11,12,13,14,15,16,17,18,20,21], were selected for the three-point bending test. The specific model parameters are shown in Table 1. The test scheme is shown in Table 2. The relationship between the geogrid and mortar is simplified according to the method of Huang Mingping [22], This method gives a parallel bond model between ‘geogrid-mortar’ particles. 

## 4. Results and Discussion

**Figure 7:**
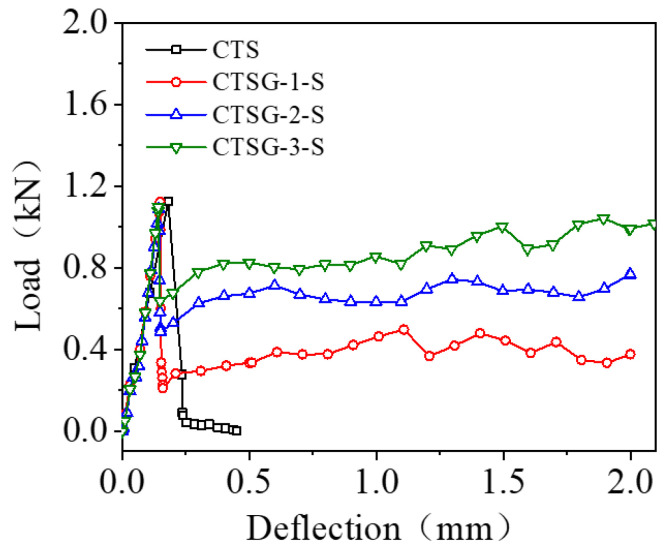
Load–deflection diagram of single-layer geogrid CTSG.

Figure 7 shows the load–deflection curves for single-layer CTSG specimens with different geogrid stiffnesses. It can be observed that the load measured from the actuator quickly reaches the peak load P1 with the increase in deflection; after that, brittle failure occurs and the bearing capacity drops to 0. The peak load P1 of the CTSG specimen is close to that of the CTS specimen. With the increase in deflection, although the load on the CTSG specimen also decreases rapidly, the geogrid limits the further development of the cracks, so that the load decreases to a certain value and then increases slowly. The geogrid reinforcement can effectively prevent the specimen from undergoing brittle failure. Compared with CTSG-1-S, the CTSG-3-S specimen has a smaller decrease in load after the peak value, and then the subsequent load increases faster. The value of the load drop and subsequent increase after the peak in CTSG-2-S is between the above two working conditions.

**Figure 8:**
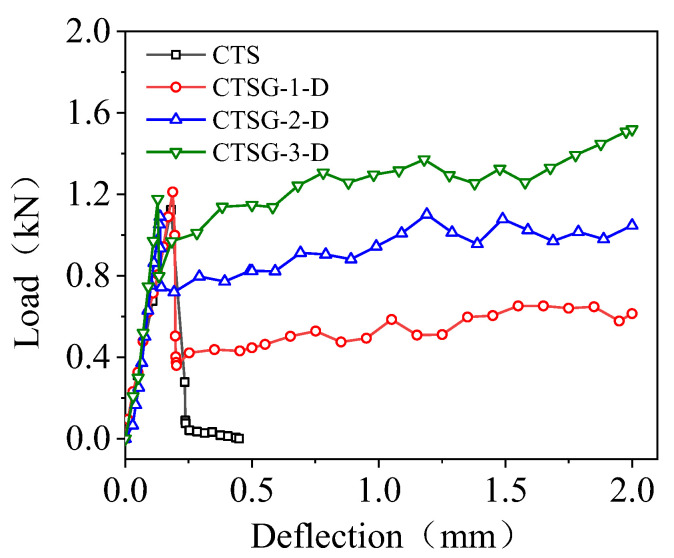
Load–deflection diagram of double-layer geogrid CTSG.

Figure 8 shows the load–deflection curves for double-layer CTSG specimens with different geogrid stiffnesses. It can be seen from the figure that the load–deflection relationship of double-layer CTSG specimens changes with deflection and stiffness in a similar way to single-layer CTSG specimens. By comparing with Figure 7, it can be found that increasing the number of layers can effectively limit the drop in load after the peak value and increase the load recovery rate in the load recovery stage under the same geogrid stiffness. For the CTSG-3-D specimen with the highest stiffness and the most reinforcement layers of geogrids, the load applied to the sample can be restored to above the peak point P1 at a large deflection.

**Figure 9:**
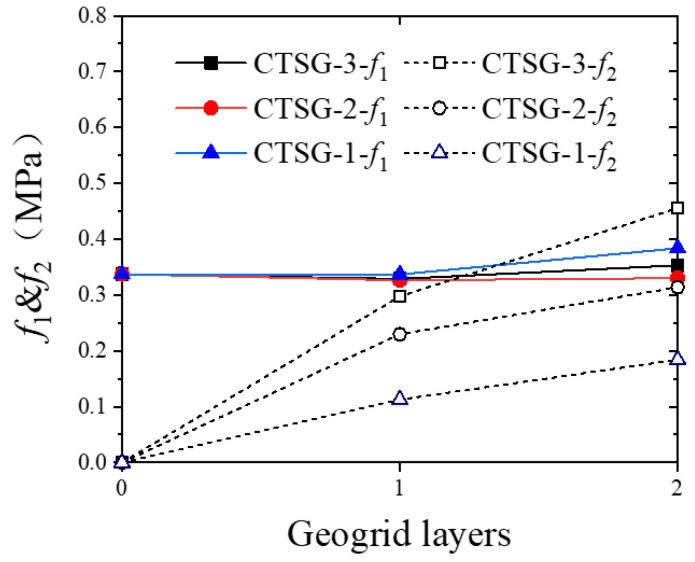
Reinforced layers-bending strength *f*_1_, *f*_2_ (MPa).

Figure 9 shows the relationship between flexural strength and the number of reinforcement layers for CTSG specimens with different geogrid stiffnesses. It can be observed that the number of reinforcement layers and the geogrid stiffness have little effect on the first peak strength *f*_1_, but a significant effect on the second peak strength *f*_2_. The quantitative comparison of *f*_2_ for each specimen indicates that for single-layer reinforcement specimens, the *f*_2_ of CTSG-3-S is 184.84 kPa higher than that of CTSG-1-S and 67.87 kPa higher than that of CTSG-2-S; for double-layer reinforcement specimens, the *_f_*_2_ of CTSG-3-D is 271.49 kPa higher than that of CTSG-1-D and 141.52 kPa higher than that of CTSG-2-D. The *f*_2_ value increases significantly with the increase in geogrid stiffness, and the increase in the number of reinforcement layers will enhance the effect above, resulting in a larger growth rate. This is because in the early stage of loading, the cement mortar of the specimen has not been cracked yet, and the geogrid and cement mortar undergo coordinated deformation under external forces. The load is mainly controlled by the cement mortar part which occupies a higher stiffness, leading to the similar *f*_1_ values for different specimens. However, when the specimen reaches *f*_2_, corresponding to a deflection of 2.0 mm or L/150, the cement mortar in the cross-section has cracked at this point. The geogrid plays its reinforcing role, and as a result, the stiffness and number of layers of the geogrid significantly affect the value of *f*_2_.

**Figure 10:**
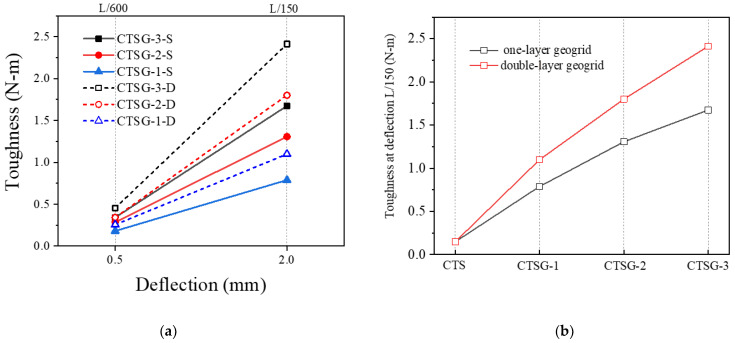
Comparison of toughness of CTSG specimens under different working conditions (**a**) The change in toughness of different specimens with deflection. (**b**) Comparion of toughness of different stiffness geogrid specimens.

The Figure 10a shows the deflection–toughness curve for CTSG specimens. It can be observed that the toughness values of the CTSG specimens under different conditions increase differently as the deflection of the specimens increases from L/600 to L/150. The specimens with higher stiffness of the geogrid and more layers of reinforcement experience a greater increase in toughness. Figure 10b shows the variation in toughness of the specimens at a deflection of L/150. It can be seen that increasing the number of geogrid layers and the stiffness of the geogrid can improve the toughness of the CTSG specimens, and the growth rate of toughness varies with different reinforcement conditions. Compared to CTSG-1, the toughness of single-layer or double-layer CTSG specimens (CTSG-2) increased by 51.9% and 70.2%, respectively. Compared to CTSG-1, the toughness of single-layer or double-layer CTSG specimens (CTSG-3) increased by 88.6% and 131.4%, respectively. It is evident that increasing stiffness significantly enhances the toughness of CTSGs, but the growth rate shows a decreasing trend. Compared to CTSG-1-S, the toughness of double-layer CTSG specimen (CTSG-1-D) is 39.1% higher, while the toughness of CTSG-3-D is 44.1% higher than that of CTSG-3-S. This indicates that increasing the number of reinforcements is more beneficial when the stiffness of the geogrid is greater.

**Figure 11:**
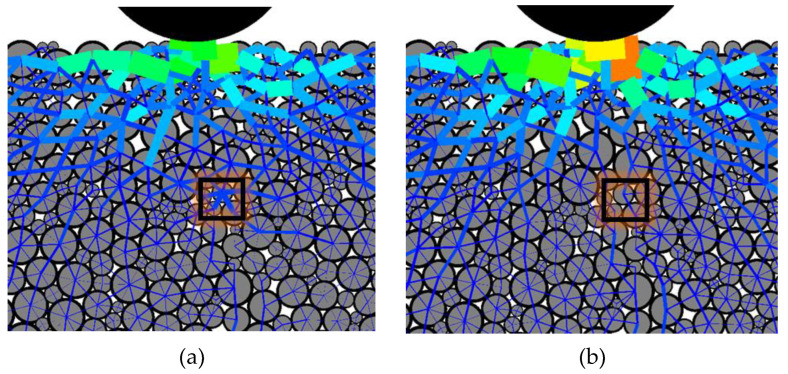
Specimen crack tip force chain amplification diagram. (**a**) The state of mortar inside the box before cracking, (**b**) The state of mortar inside the box after cracking.

Taking CTSG-2-S as an example, Figure 11 displays an amplified view of the force chain at the crack tip of the specimen. The force chain in the box in Figure 11a is complete, and its thickness is conspicuously greater than that of the surrounding particles. It is observed that the force chain between the cement particles at the crack tip is significantly greater than that of the surrounding particles, leading to stress concentration. Subsequently, as the loading progresses, the force chain in the box fractures, as shown in Figure 11b, causing the particle force chains in the box to fracture, and consequently extending the crack upwards.

**Figure 12:**
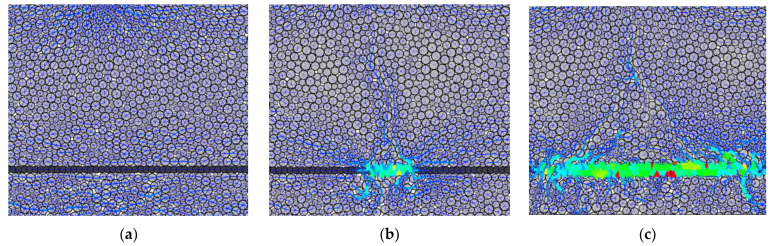
Force chain diagram of ctsg specimen in bending process (local amplification near geogrid). (**a**) before cracking, (**b**) Initial cracking, (**c**) cracking development.

It can be seen in Figure 12, during the initial loading stage, that the specimen remains uncracked, and the force chains between particles are relatively weak, with slightly larger transverse force chains across the bottom. At this stage, the forces between the geogrid particles and cement particles are relatively small and exhibit a nearly independent relationship. As the loading progresses, the bond between the cement particles across the bottom is broken, and cracks begin to appear. Subsequently, the geogrid particles and cement particles exhibit significant compressive stress, and crushed cement mortar can be observed on top of the geogrid at mid-span. With further loading, the area of the geogrid under stress increases, and more cracks are generated. Notably, there is a greater contact force between the cement particles at the end of the geogrid under tension.

**Figure 13:**
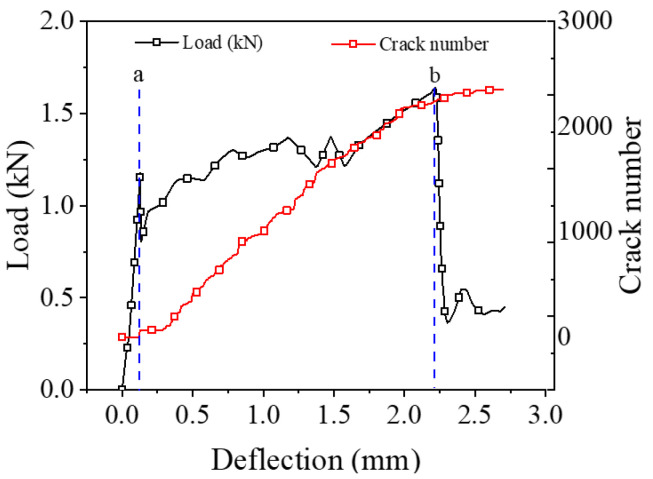
Crack number and load–deflection diagram of CTSG-3-D specimen.

The distribution and evolution of cracks under different working conditions are different. This paper studies the bending response of CTSG specimens by analyzing the change in the number of cracks during the bending process. Figure 13 shows the evolution of load and crack number with deflection. According to the two extreme points of the load–deflection curve, the bending failure process of the reinforced specimen can be divided into three stages: the pre-failure stage, the crack development stage and the residual failure. In the pre-failure stage, the number of cracks remained small, and the specimen was intact. At this time, the load increases linearly with the deflection. When the deformation reaches the first extreme point, the specimen cracks, the number of cracks increases suddenly, and the load decreases sharply. As the deflection continues to increase, the specimen enters the crack development stage, during which the bending cracking continues, the number of cracks increases steadily, and the load increases gradually. When the deflection reaches the second extreme point, the specimen is further destroyed. At this time, the number of cracks is basically stable, the bearing capacity of the specimen drops sharply.

**Figure 14:**
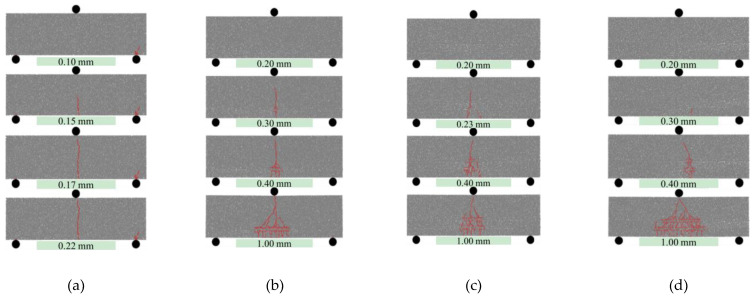
Crack development of different specimens. (**a**) CTS. (**b**) CTSG-2-S. (**c**) CTSG-2-D. (**d**) CTSG-3-D.

In order to visually display the crack state of CTSG specimens and show the fracture path, this study uses red line segments to mark the crack surface. Figure 14 shows the crack evolution process of several specimens. The CTS specimen presented brittle failure. After the appearance of the crack, it rapidly develops and penetrates the cross-section, with a single fracture. For the CTSG specimen, the crack path change from a single penetrating crack to multiple cracks at mid-span, and the range of crack distribution significantly expands, showing a triangular shape, and the failure mode exhibits ductile fracture. This is because the anchoring effect between the geogrid and the cement mortar and the anchoring effect limit the upward development of the main crack and prevent the specimen from fracturing. As the test continues, the geogrid is subjected to greater tensile stress, and the tensile stress is transmitted to the cement mortar through the anchoring effect, which leads to more cracks in the cement mortar, resulting in the appearance of multiple cracks. Comparing Figure 14b–d, it can be seen that with the increase in the geogrid stiffness and number of layers, the range of crack distribution expands. This is because the increase in geogrid stiffness and number of layers can enhance the anchoring effect between the geogrid and the cement mortar, thereby strengthening the anchor effect.

**Figure 15:**
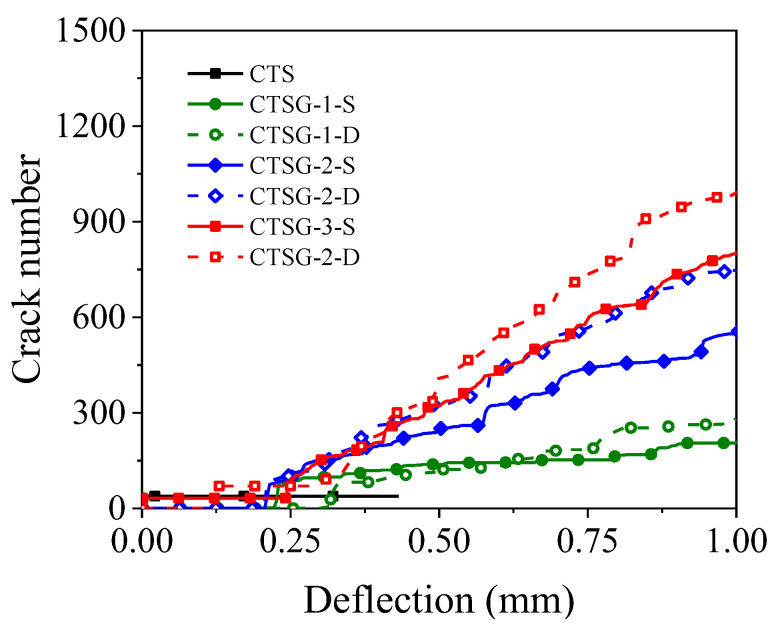
The variation in the number of cracks with deflection in different working conditions.

Figure 15 shows the variation in the number of cracks with deflection. It can be observed from the figure that the number of cracks in the CTS specimens increases slightly in the early deflection stage and then remains unchanged, while the number of cracks in the CTSG specimens continues to increase during the bending process. This is because brittle failure occurs under small deflection in the CTS specimen, and the crack development stops. In contrast, the CTSG specimens did not fail after cracking due to the anchoring effect between the geogrid and cement mortar. The geogrid transmits tensile force through anchorage, making the cement mortar participate in bearing until reaching the ultimate tensile strain, leading to failure and exhibiting a wider distribution of cracks. In addition, the higher the geogrid stiffness and the more reinforcement layers, the faster the increase rate of crack number after cracking. This is because the geogrid with higher stiffness and more layers has a stronger bonding and anchoring effect, which can drive more cement mortar to participate in bearing. In summary, the geogrid changes the failure mode of the specimens through anchorage with the mortar, and the stiffness and number of reinforcement layers of the geogrid affect the crack development rate and distribution pattern.

**Figure 16:**
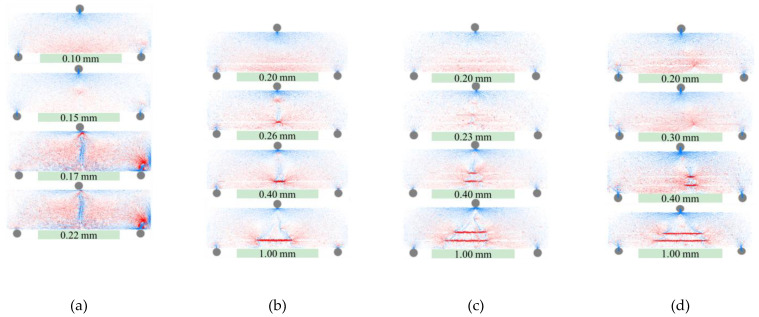
The evolution of internal force with loading for different working conditions. (**a**) CTS. (**b**) CTSG-2-S. (**c**) CTSG-2-D. (**d**) CTSG-3-D.

Figure 16 shows the internal force of the specimens at different deflection levels. The red lines represent tensile stresses, blue lines represent compressive stresses. The thickness of the line represents the value of the internal force, in which the thick represents large and the thin represents small. Figure 16a shows the internal force diagram of the CTS specimen, which is in a state of tension at the bottom and compression at the top before cracking. After cracking, the internal force decreases significantly, and a red stress concentration zone appears at the crack tip. Under the action of this stress concentration, cracks continue to develop until the section is completely ruptured (the line thickness is affected by normalization after specimen failure). Figure 16b–d show the internal force evolution of the CTSG specimens. Before cracking, the geogrid and cement mortar undergo coordinated deformation. Due to the lower stiffness of the geogrid, the tensile stress on the geogrid is relatively small. After cracking, the cement mortar in the crack fails, resulting in the geogrid experiencing a concentration of tensile stress. As a result, radial tensile stress lines can be observed connecting the surrounding cement mortar. As deflection increases, cracks continue to develop, causing the geogrid’s tensile stress concentration range to expand to both sides and the radial stress line to move towards both ends of the specimen. The radial stress lines are caused by the anchoring effect between the geogrid and cement mortar, which transmits tensile stress from the cement mortar to the mortar after cracking. As can be seen in Figure 16c,d, both layers of the geogrid in the double-layer CTSG beam specimens exhibit concentrated tensile stress, resulting in four radial stress line regions. It can be observed that the lower layer of the geogrid has a longer tensile stress concentration zone. This is attributed to the greater deformation of the lower layer of the geogrid under bending of the specimen, resulting in a greater tensile stress and a greater tensile stress transmitted to the surrounding mortar.

**Figure 17:**
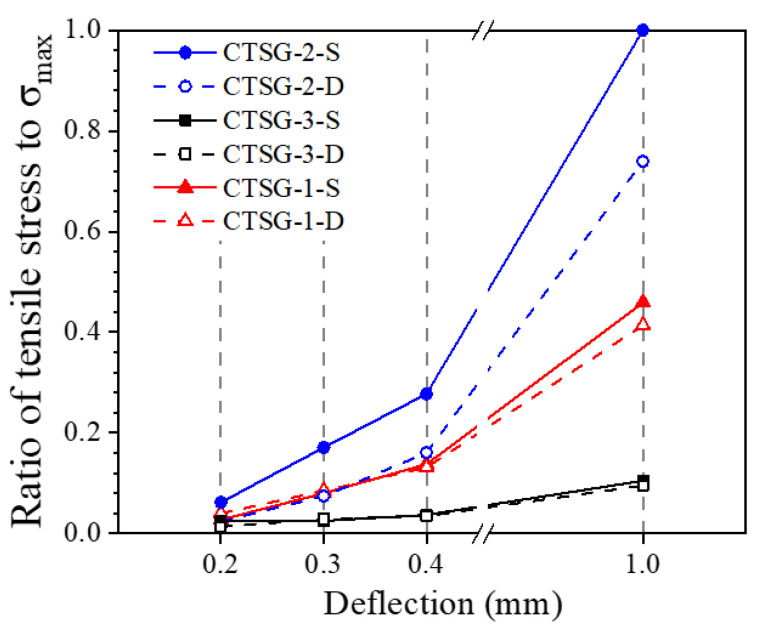
Stress–deflection diagram of geogrid.

The average internal contact force (tension) of the geogrid at different deflections is quantified and normalized according to the internal forces of the CTSG-3-S specimen, as shown in Figure 17. It can be seen from the figure that as the deflection of the specimen increases, the internal force tends to increase linearly. At the initial stage of loading, the value of geogrid forces in CTSG specimens with different stiffness and layers are similar; this is because the deformation of the geogrid is small, and the load is borne by the stiffer cement mortar. As the loading continues, the internal force of the geogrid starts to be stimulated. The internal force growth rate with a larger geogrid stiffness is faster. The geogrid internal force of CTSG-3-D at a deflection of 1.0 mm is 26.1% smaller than that of CTSG-3-S, the geogrid internal force of CTSG-2-D is 9.8% smaller than that of CTSG-3-S and the geogrid internal force of CTSG-1-D is 9.1% smaller than that of CTSG-1-S. This indicates that the average stress of the double-layer CTSG specimen is smaller than that of the single-layer CTSG specimen, and the larger the stiffness of the geogrid, the more significant this difference, mainly because the average position of the double-layer reinforced geogrid is closer to the compression zone.

To investigate the evolution of normal contact force chain in different specimens, diagrams of the normal contact force chain under different loading conditions are plotted. The Fourier function proposed by ROTHENBURG [23] is used to describe the distribution and anisotropy of contact force chain.

(3) $$f_{n}\left(\theta \right)=f_{0}\left\{1+a_{n}cos\left[2\left(\theta -\theta_{n}\right)\right]\right\}$$

In the equation,$\;f_{n}\left(\theta \right)$ represents the mean normal contact force in the angular interval, N; $f_{0}$ represents the mean value of all normal contact forces, N; $a_{n}$ represents the Fourier fitting coefficient and $\theta_{n}$ represents the main direction of anisotropy.

**Figure 18:**
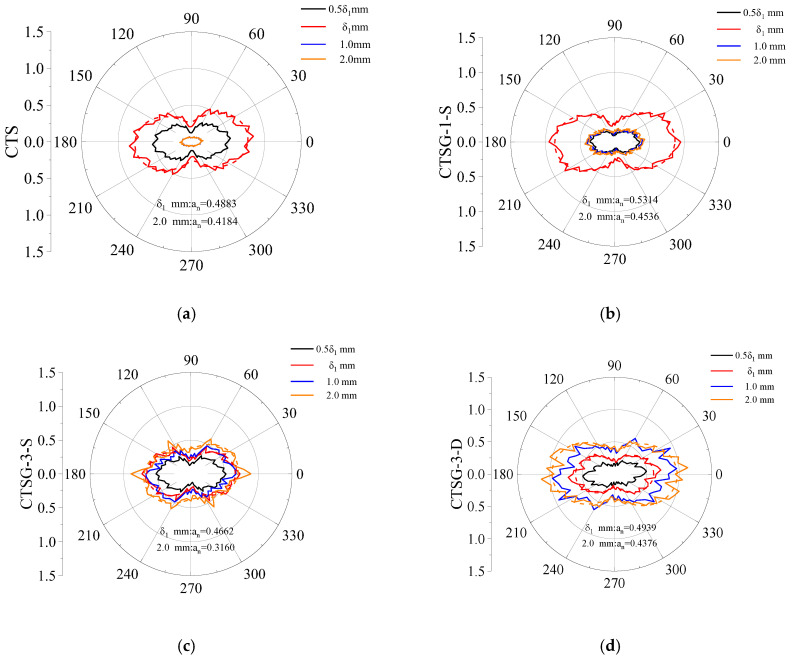
Composition diagram of normal contact force chain. (**a**) CTS. (**b**) CTSG-1-S. (**c**) CTSG-3-S. (**d**) CTSG-3-D.

The data in the figures have been normalized using the maximum component of the normal contact force distribution when the deflection of CTSG-1-S reaches δ1. δ1 is the deflection corresponding to the first peak value point of each specimen. The dashed lines in the figures represent the Fourier fitting curves. As shown in Figure 18, the normal contact force of each specimen has a similar distribution, and they are all of a “peanut” shape. The normal contact force in the horizontal direction is significantly larger than that in the vertical direction, thereby resisting the tensile force generated by the bending moment on the lower side of the sample. The normal contact force distribution of the CTS and CTSG specimens are similar when the deflection of the specimen reaches the first cracking deflection *δ*_1_, because the specimen has not yet cracked and the geogrid has little effect at this time. After the deflection of the CTS specimen reaches δ1, the specimen is destroyed, the area surrounded by the normal contact force distribution diagram decreases rapidly and it no longer changes with the increase in the deflection. Comparing CTSG-1-S and CTSG-3-S, when the stiffness of the geogrid is low, the normal contact force of the specimen reaches its maximum when the deflection is δ1, and then the area surrounded by the normal contact force distribution graph is significantly reduced. With the further increase in the deflection, although the area increases, it still cannot reach its maximum value. Under high stiffness, the normal contact force of the specimen decreased after the deflection exceeds δ1, but could be restored to a level higher than that at δ1 due to a stronger reinforcement effect.

The Fourier fitting coefficient a_n_ decreased with increasing geogrid stiffness (which means anisotropy of mortar decreased). Comparing CTSG-3-S and CTSG-3-D, it can be seen that the contact force of double-layer reinforced specimens decreased smaller at cracking and had a greater contact force value when deflection come to 2 mm. The Fourier fitting coefficient a_n_ of double-layer reinforced specimens increased, as the double-layered reinforcement increased the range of the mortar tensile area (as shown in Figure 18d), resulting in a more significant increase in the contact force in the horizontal direction inside the mortar, enhancing the anisotropy.

## 5. Conclusions

To investigate the influence of the number of layers and stiffness of geogrids on geogrid reinforced cement-treated sand (CTSG) and analyze the mesoscopic mechanism of their impact on bending resistance, this study conducted laboratory three-point bending tests on CTSG specimens with different layers of geogrid reinforcement, and a series of DEM simulation tests are conducted to supplement the effect of geogrid stiffness. The experimental results compared the flexural response process and mesoscopic parameters evolution of CTSG under different conditions, leading to the following conclusions.

Geogrids can improve the flexural performance of CTSGs, and the increase in geogrid stiffness and layers has a promoting effect on this improvement. The failure process of CTSG specimens can be divided into three stages: pre-fracture, crack development and residual failure, exhibiting good ductility. Geogrid reinforcement can enhance the flexural strength and toughness of CTSG specimens. The increase in geogrid stiffness and layers can suppress the strength loss of the first peak strength, promote load recovery and make the specimen more resilient.

Geogrids have a significant impact on the crack propagation rate and distribution of CTSG specimens. Once CTS specimens crack, the main crack quickly penetrates the cross-section, and the number of cracks is small when the specimen fails. After cracking, due to the anchoring effect of the geogrids in CTSGs, the main crack develops slowly with the increase in deflection. At the same time, geogrids transmit tensile stresses to the surrounding mortar, causing the mortar to produce multiple fine triangular cracks in the area of tensile stress concentration. The increase in geogrid stiffness and layers can strengthen the anchorage effect and increase the crack distribution area and total number of cracks in the specimen.

There is a marginal effect of changing the number and stiffness of geogrids on the toughness improvement of CTSG specimens. The toughness growth rate of CTSG specimens decreases as the stiffness of the geogrids increases. The greater the stiffness of the geogrids, the greater the toughness growth rate of CTSG specimens when the number of reinforcements is increased. When the stiffness of the geogrid is low, improving the stiffness has a better effect on the toughness improvement of CTSG specimens, and the improvement of single-layer CTSG specimens is greater than that of double-layer CTSG specimens. When the stiffness of the geogrid is high, the benefit of increasing the number of reinforcements is higher. CTSG-1-D has a toughness value 39.1% higher than CTSG-1-S, while CTSG-3-D has a toughness value 44.1% higher than CTSG-3-S.

Increasing the number of layers of the geogrid has a promoting effect on the anisotropy of the normal contact force inside the CTSG specimen, while increasing the stiffness of the geogrid shows an inhibitory effect. The reason may be that increasing the number of layers of geogrid will increase the tensile area of the specimen when it is bent, and the internal contact force of the mortar will increase more significantly in the horizontal direction, thereby enhancing the anisotropy.

## Figures and Tables

**Table 1:** Numerical test model parameter table.

Parameter	Cement Mortar	Geogrid
Density (kg/m^3^)	2660	1350
Normal stiffness (N/m)	5 × 10^8^	1 × 10^7^, 5 × 10^7^, 1.0 × 10^8^
Normal-tangential stiffness ratio (N/m)	1.2	1.2
Parallel bond normal stiffness (N/m^3^)	2 × 10^11^	1.2 × 10^10^, 6 × 10^10^, 1.2 × 10^11^
Parallel bond tangential stiffness (N/m^3^)	1.67 × 10^11^	1 × 10^10^, 5 × 10^10^, 1 × 10^11^
Tensile strength (N/m^2^)	7 × 10^5^	1 × 10^8^
Cohesion (N/m^2^)	7 × 10^5^	1 × 10^8^

**Table 2:** Test plan.

Specimen Code	Geogrid Stiffness (N/m)	Geogrid Layers
CTS	——	——
CTSG-1-S	1 × 10^7^	One-layer
CTSG-2-S	5 × 10^7^	One-layer
CTSG-3-S	1 × 10^8^	One-layer
CTSG-1-D	1 × 10^7^	Double-layer
CTSG-2-D	5 × 10^7^	Double-layer
CTSG-3-D	1 × 10^8^	Double-layer

## Data Availability

Not applicable.

## References

[1] Zhang Z., Guo X. (2021). Review of geogrid and geotextile reinforced foundation. Subgrade Eng..

[2] Hadi M., Al-Hedad A. (2020). Flexural fatigue behaviour of geogrid reinforced concrete pavements—ScienceDirect. Constr. Build. Mater..

[3] Liu Y., Wang L. (2021). Effect of geogrid on basic mechanical properties of macroporous concrete. Sichuan Build..

[4] Siva Chidambaram R., Agarwal P. (2015). Flexural and shear behavior of geo-grid confined RC beams with steel fiber reinforced concrete. Constr. Build. Mater..

[5] Siva Chidambaram R., Agarwal P. (2014). The confining effect of geo-grid on the mechanical properties of concrete specimens with steel fiber under compression and flexure. Constr. Build. Mater..

[6] El Meski F., Chehab G.R. (2014). Flexural Behavior of Concrete Beams Reinforced with Different Types of Geogrids. J. Mater. Civ. Eng..

[7] Abu-Farsakh M.Y., Chen Q. (2011). Evaluation of geogrid base reinforcement in flexible pavement using cyclic plate load testing. Int. J. Pavement Eng..

[8] Rochanavibhata U., Jamnongwong M., Chuenjaidee S., Jamsawang P., Chen X.B. (2020). Flexural Behavior of Compacted-Cement Sand Reinforced with Geogrid. Key Eng. Mater..

[9] Al-Hedad A.A., Hadi M.N.S. (2019). Effect of Geogrid Reinforcement on the Flexural Behaviour of Concrete Pavements.

[10] Wang L., Zhang Y., Forward B., Liu H. (2018). Centrifugal Model test of reinforced soil retaining wall under different reinforcement stiffness. J. Yangtze River Acad. Sci..

[11] Cundall P.A., Strack O.D.L. (1980). A discrete numerical model for granular assemblies. Géotechnique.

[12] Lan H., Martin C.D., Hu B. (2010). Effect of heterogeneity of brittle rock on micromechanical extensile behavior during compression loading. J. Geophys. Res..

[13] Lee H., Ji H.K., Kim K.H., Cho H.C. (2008). Application of DEM model to breakage and liberation behaviour of recycled aggregates from impact-breakage of concrete waste. Min. Eng..

[14] Tan X., Li W., Zhao M., Tam V.W.Y. (2019). Numerical Discrete-Element Method Investigation on Failure Process of Recycled Aggregate Concrete. J. Mater. Civ. Eng..

[15] Wang P., Gao N., Ji K., Stewart L., Arson C. (2020). DEM analysis on the role of aggregates on concrete strength. Comput. Geotech..

[16] (2012). Standard Test Method for Flexural Performance of Fiber-Reinforced Concrete (Using Beam With Third-Point Loading).

[17] Miao C., Jia Y., Zhang J., Zhao J. (2020). DEM simulation of the pullout behavior of geogrid-stabilized ballast with the optimization of the coordination between aperture size and particle diameter. Constr. Build. Mater..

[18] Song Z., Konietzky H., Herbst M. (2019). Three-dimensional particle model based numerical simulation on multi-level compressive cyclic loading of concrete. Constr. Build. Mater..

[19] Mohammadinia A., Oskooei P.R., Arulrajah A. (2021). Discrete element modeling of cemented recycled concrete aggregates under unconfined and k0 loading conditions. Transp. Geotech..

[20] Chen J., Zheng X., Li H. (2011). Two-dimensional discrete-continuous coupling simulation of reinforced soil retaining wall on soft soil foundation. J. Civ. Eng..

[21] Chen C., Duan Y., Rui R. (2021). Research on single and double geogrid reinforced ballast based on pull-out test and discrete element simulation. Geotech. Mech..

[22] Huang P., Pan X., Niu Y. (2022). Study on ultimate bearing capacity of reinforced concrete beams based on discrete element. Eng. Mech..

[23] Hua W., Xiao Y., Wang M. (2021). Discrete element simulation study on the influence of gradation and particle shape on the shear performance of subgrade filling with complex accumulation. J. Cent. South Univ. Nat. Sci. Ed..

